# The Future of the Poor-Law Hospital

**Published:** 1920-10-16

**Authors:** 


### The Future of the Poor-Law Hospital.

Under this title an interesting paper was read by Mr. Gladstone Walker, Clerk to the Newcastle Board of Guardians, at the recent Poor-Law Conference at Keswick. The following is a condensed report of his remarks:

Mr. Walker said that since 1894 the most important event affecting Boards of Guardians is the Ministry of Health Act, 1919. The proposals of the Consultative Council which the Minister of Health has appointed involve the transfer of Poor-Law infirmaries, and in many cases buildings now used for the sick, to the Health Authority. The Poor Law, which provides more beds for the sick than all the voluntary and municipal hospitals added together, and is an existing organisation capable of extension, cannot be uprooted without confusion and injury to the sick poor. The union areas, subject to a few alterations, are convenient and adaptable, and will economically fit any redistribution of the health services. With the area of administration the future of the Poor-Law hospital is bound up. The county and municipal authorities provide for their respective areas some 40,000 hospital beds in all. There are 1,840 local sanitary authorities and sixty-two County Councils in England and Wales. These bodies provide almost solely fever and smallpox beds. In Northumberland and Durham it has been computed that there are twenty-eight hospitals with 730 beds reserved for smallpox, and remaining empty—a monument to the disease! On the other hand, there is a monument to the charitable public in 40,573 beds, with a huge waiting-list, running to 3,000 in the counties just named.

The point he wished to emphasise was that, while the 1,900 authorities authorised to provide hospitals for the sick, as well as for infectious cases, average only twenty beds each, the 637 Poor-Law authorities, without specific legal authority, but acting purely for the public health, have provided 94,000 hospital beds, an average of 150 for each union area. More is involved in the recent report than the taking over of the Poor-Law hospitals. Some form of State medical service is foreshadowed.

The Guardians must help to break down the prejudice which still prevents the sick from making general use of the Poor-Law hospitals. Since voluntary hospitals are full, and the fees of nursing homes prohibitive, a large section of the public is now technically destitute in the legal sense. In regard to the future, he thought that the medical service would co-operate with the Government to bring the treatment of the sick poor under one authority, to which the health service of the Poor Law would be transferred. The financial difficulties of the voluntary hospitals and the valuable clinical experience to be gained only in Poor-Law infirmaries and the sick wards of Poor-Law institutions are fully appreciated by the medical service. But if the treatment of the sick poor were transferred Boards of Guardians would certainly perish.

